# A Spin-Labeled Derivative of Gossypol

**DOI:** 10.3390/molecules29204966

**Published:** 2024-10-21

**Authors:** Andrey V. Stepanov, Vladimir N. Yarovenko, Darina I. Nasyrova, Lyubov G. Dezhenkova, Igor O. Akchurin, Mickhail M. Krayushkin, Valentina V. Ilyushenkova, Andrey E. Shchekotikhin, Evgeny V. Tretyakov

**Affiliations:** 1 N.D. Zelinsky Institute of Organic Chemistry, Leninsky Ave. 47, Moscow 119991, Russia; av_stepanov@mail.ru (A.V.S.); yarovladimir@yandex.ru (V.N.Y.); nasyrova-01@mail.ru (D.I.N.); v-ilyushenkova@ioc.ac.ru (V.V.I.); 2 Gause Institute of New Antibiotics, Bolshaya Pirogovskaya St. 11, Moscow 119021, Russia; dezhenkovalg@yahoo.com (L.G.D.); akchurin.i.o@muctr.ru (I.O.A.); shchekotikhin@mail.ru (A.E.S.); 3 D. Mendeleev University of Chemical Technology of Russia, Miusskaya Sq. 9, Moscow 125047, Russia

**Keywords:** polyphenols, gossypol, nitroxide radicals, Schiff bases, X-ray, antiproliferative activity, radical biological chemistry

## Abstract

Gossypol and its derivatives arouse interest due to their broad spectrum of biological activities. Despite its wide potential application, there is no reported example of gossypol derivatives bearing stable radical functional groups. The first gossypol nitroxide hybrid compound was prepared here via formation of a Schiff base. By this approach, synthesis of a gossypol nitroxide conjugate was performed by condensation of gossypol with a 4-amino-TEMPO (4-amino-2,2,6,6-tetramethylpiperidin-1-oxyl) free radical, which afforded the target product in high yield. Its structure was proven by a combination of NMR and EPR spectroscopy, infrared spectroscopy, mass spectrometry, and high-resolution mass spectrometry. In addition, the structure of the gossypol nitroxide was determined by single-crystal X-ray diffraction measurements. In crystals, the paramagnetic Schiff base exists in an enamine–enamine tautomeric form. The tautomer is strongly stabilized by the intra- and intermolecular hydrogen bonds promoted by the resonance of π-electrons in the aromatic system. NMR analyses of the gossypol derivative proved that in solutions, the enamine–enamine tautomeric form prevailed. The gossypol nitroxide at micromolar concentrations suppressed the growth of tumor cells; however, compared to gossypol, the cytotoxicity of the obtained conjugate was substantially lower.

## 1. Introduction

In recent years, organic radicals have been actively studied [1], with most attention being paid to applied aspects of this research [2]. Successful uses of stable radicals in various fields of physics, chemistry, and medicine have been described. For example, they are applied in organic synthesis of various substances and materials [3], including natural compounds [4], organic batteries [5,6], devices for converting solar energy into electrical energy, and powerful antioxidant and radioprotective agents [7,8,9]. The sensitivity of EPR spectra of organic paramagnets to a local environment also makes them valuable probes for studying complex molecular systems [10]. In addition, profluorescent compounds with a radical moiety, which are used as luminescent probes [11] or fluorescent antibiotics [12], are a unique tool for elucidating modes of action of antibiotics on bacteria. Hybrid compounds containing new types of spin-labeled profluorophores based on 3-acyl-2-hetarylchromones, which are capable of responding to both light irradiation and magnetic fields, have been developed recently [13,14].

The basis for the wide range of applications of stable organic radicals is the possibility of preparation of their various polyfunctional derivatives. In this regard, convenient objects are nitroxide radicals, which are compatible with a wide variety of synthetic reactions [15], including Diels–Alder [16], Friedel–Crafts [17], Michael-type additions [18], Pd-catalyzed cross-coupling [19,20,21], click chemistry [22,23,24], carbodiimide coupling [25], and many others [26]. The developed chemistry of nitroxides shows that syntheses in their presence can be surprisingly facile and predictable. This advantage allows for their use in functionally oriented syntheses of various nitroxides. Very recently, synthesis of “sterically shielded” nitroxides has been developed; in biological samples and in cells, they possess much higher stability than conventional tetramethyl-substituted nitroxides [27]. A method for the preparation of stable cyclic α-hydrogen nitroxides [28] has been proposed. A new class of trityl nitroxide biradicals [29,30] and nitroxides [31] has been described as polarizing agents for high-field dynamic nuclear polarization (DNP)/NMR spectroscopy of complicated biomolecules. To date, thousands of nitroxide radicals have been prepared and characterized, and some of them have found applications in various areas of biology and medicine [32,33,34]. In particular, rapid growth of interest in the role of “oxidative stress” in pathological processes has drawn attention to drugs that can prevent the formation of reactive oxygen species or accelerate their inactivation [35,36]. In this regard, attention is given to the stable nitroxides that can act as antioxidants that protect cells and tissues from oxidative processes [37,38]. It has been shown that nitroxide radicals, via modifying oxidative stress and changing the oxidative status of tissues, are capable of regulating many metabolic processes, e.g., activation of apoptosis of tumor cells [39] and prevention of damage to neurons under powerful infrasound exposure [40].

The biological activity of nitroxides can depend not only on properties of the paramagnetic moiety but also on the presence of pharmacophoric groups in them or in their metabolites. The introduction of nitroxide groups into biologically active compounds is one of the ways to modulate their pharmacological properties. In this regard, gossypol (**1**, 2,2′-bis(1,6,7-trihydroxy-5-isopropyl-3-methyl-8-formylnaphthalene)—a large-tonnage byproduct of cotton processing—seems to be an attractive natural scaffold for conjugation with nitroxides. This polyphenol substance possesses a broad spectrum of bioactivities and is capable of forming complex compounds [41].

The unique structural features of this polyfunctional compound and chemical properties of gossypol (**1**) allow them to form fairly stable bonds with various proteins and to be easily integrated into phospholipid layers of cytoplasmic membranes. Owing to such interactions, gossypol blocks the activity of some enzymes involved in various metabolic pathways of plant and animal cells [42,43]. Reviews [44,45,46] describe its antifertility, antimalarial, antifungal, antiviral, and antiparasitic properties. As a polyphenol, it has a strong antioxidant effect [47,48]. Gossypol is being studied as a SARS-CoV-2 inhibitor that affects RNA-dependent RNA polymerases [49]. Gossypol derivatives have been shown to be promising agents for the development of drugs against leukemia, lymphoma, colon carcinoma, breast cancer, fibroids, prostate cancer, and other malignant tumors [50,51].

Much attention is currently being focused on the synthesis and investigation of imine derivatives of gossypol to find novel biologically active substances [46,52,53,54,55]. The literature data show that Schiff bases of gossypol have lower toxicity and retain the biological activity of the parental molecule. Therefore, imines of gossypol could find an application as therapeutic agents instead of gossypol, which has some adverse effects. A recent work [56] presents the synthesis of diimines by the interaction of gossypol **1** with thiohydrazides of oxamic acids; such diimines are promising for the search for substances with pharmacological activity [57,58,59]. 

As a continuation of our earlier studies, a new Schiff base of gossypol with a 4-amino-TEMPO (4-amino-2,2,6,6-tetramethylpiperidin-1-oxyl) free radical was successfully synthesized here. Evaluation of the molecular and crystal structure of gossypol nitroxide **2**, which is in fact the first paramagnetic derivative of gossypol, was carried out, as was the first assessment of antiproliferative properties of such compounds.

## 2. Results and Discussion

**Scheme 1:**
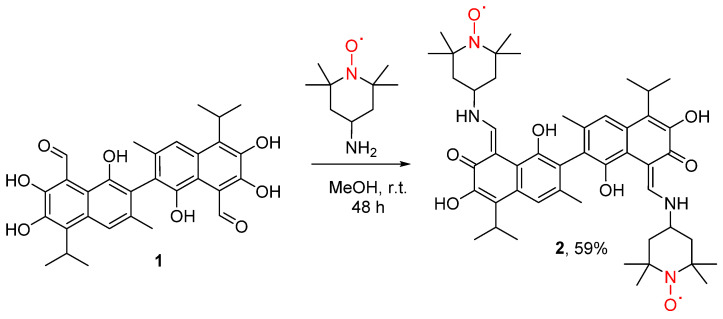
The synthesis of gossypol nitroxide **2**.

It is known that gossypol readily reacts with various amines and forms Schiff bases [52,53,54,55]. Accordingly, a reaction of **1** with two equivalents of 4-amino-TEMPO was chosen as a way to obtain a paramagnetic derivative of gossypol. The condensation proceeded smoothly in methanol and led to target derivative **2** in good yields (Scheme 1).

**Scheme 2:**
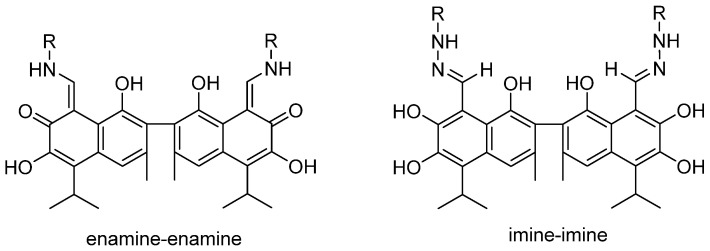
The two tautomeric forms of imine derivatives of gossypol.

It is known that imine derivatives of gossypol can exist in imine and enamine forms (Scheme 2); as a rule, products of the interaction of gossypol with amines assume an enamine–enamine form, whereas gossypol hydrazones adopt an imine–imine conformation [52,53,59]. Analogously, the formation of the imine–imine tautomeric forms has been observed after protonation of Schiff bases of gossypol [60]. Because the geometry of the gossypol derivatives has a considerable influence on their biological activity, in this work, the focus is on structural analysis of **2** in the solid phase and in solution.

**Figure 1:**
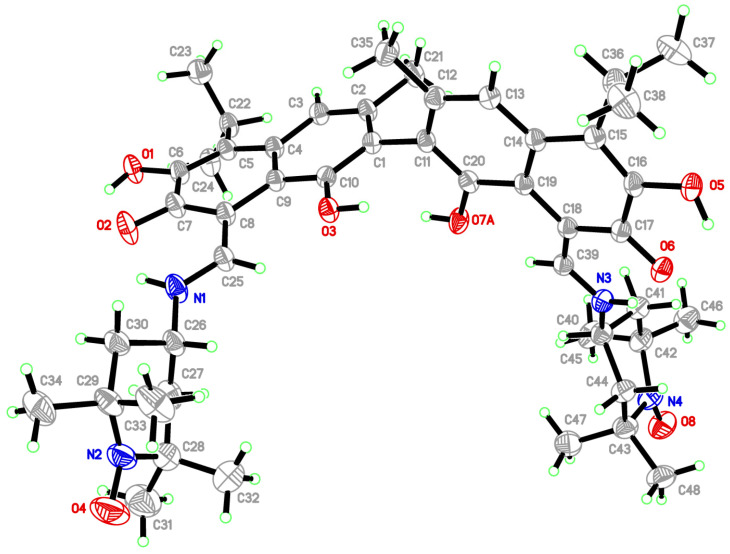
Molecular structure of gossypol nitroxide **2** and its atom-numbering schemes. Characteristics of atomic displacement are shown with a 50% probability.

Crystals of compound **2** that are suitable for single-crystal X-ray analyses were obtained by slow evaporation of an acetone/dichloromethane solution. Gossypol nitroxide **2** was found to crystallize in the trigonal *R*-3 space group (CCDC 2382454). Molecular structures of the compound are displayed as ORTEP representations in Figure 1, and selected bond lengths and angles are given in Table 1. The bond lengths in the compounds are moderate and comparable with such lengths in other imino derivatives of gossypol [61,62,63] and 2,2,6,6-tetramethylpiperidin-1-oxyls [64,65].

**Figure 2:**
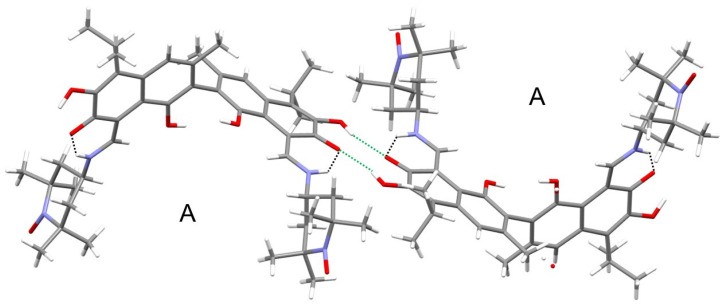
Intramolecular H-bonds (N-H⋯O: dotted black lines) and intermolecular H-bonds (O-H⋯O: dotted green lines) linking molecules of **2** into dimers {A⋯A}.

The main feature of the molecular structure of gossypol nitroxide **2** is a proton positioning in intramolecular hydrogen bridges O(2)/H/N(1) and O(6)/H/N(3) (Figure 2). In derivative **2**, the protons reside at N(1) and N(3), whereas in gossypol hydrazones, the proton is on vicinal O atoms [66]. C(7)–O(2), C(25)–N(1), and C(8)–C(25) bond lengths (as with C(17)–O(6), C(39)–N(3), and C(18)–C(39) bond lengths) (Table 1) suggest that the intramolecular hydrogen bonds that arose within the two different moieties are promoted by resonance within the π-electron aromatic system. A decrease in C(7)–O(2) and C(17)–O(6) bond lengths in crystal structures of **2** clearly showed that the structure contains carbonyl groups on naphthalene rings. This result means that in the solid state, enamine–enamine tautomeric forms exist within **2**. In these forms, parameters of intramolecular hydrogen bonds are similar to those in the gossypol Schiff bases reported earlier (Table 2) [61,62,63].

**Figure 3:**
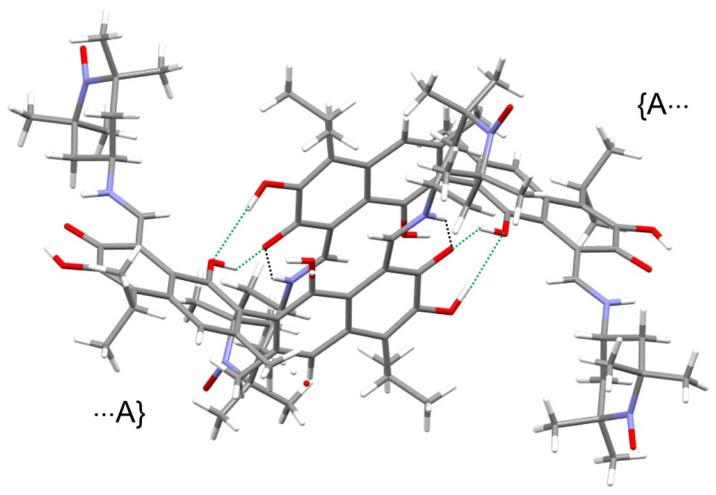
Intermolecular H-bonds (O-H⋯O: dotted green lines; N-H⋯O: dotted black lines) linking dimers {A⋯A} into chains.

**Figure 4:**
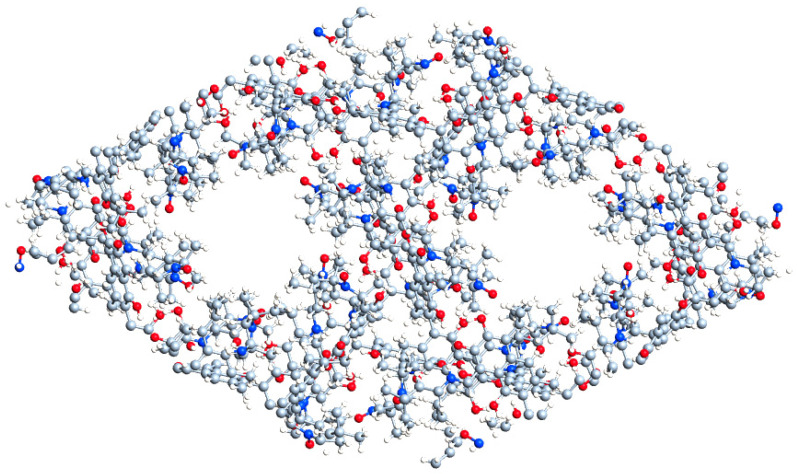
Channels in the structure of gossypol nitroxide **2**.

Gossypol nitroxide **2** manifested self-assembly within crystalline states through a combined action of different hydrogen (O⋯HO) bonds. First, the O(1)–H(1) group is involved in a relatively short intermolecular H-bond to atoms O(2) of the carbonyl group of the molecule transformed via an inversion center. These two hydrogen bonds give rise to dimers {A⋯A} (Figure 2). In turn, the dimers {A⋯A} are linked through H-bonds, thereby forming chains in which sesquiterpene systems are stacked, as illustrated in Figure 3. The chains are assembled via another type of noncovalent interactions, thus forming a channel-shaped network structure (Figure 4).

**Figure 5:**
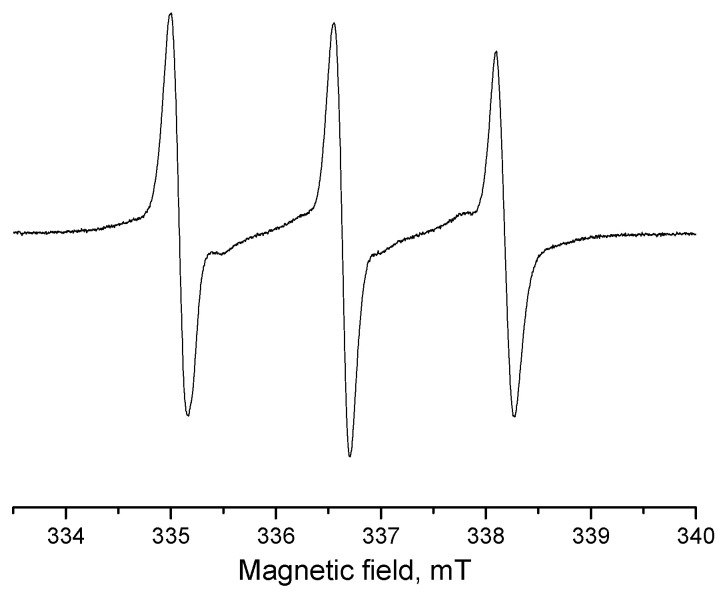
The experimental RT X-band spectrum of gossypol nitroxide **2** in toluene (10^−4^ M).

To characterize the local structure of paramagnetic parts of gossypol nitroxide **2**, we performed room temperature (RT) X-band (9.87 GHz) continuous-wave EPR experiments on 10^−4^ M solutions in toluene. Despite the biradical character of **2**, the spectrum is very similar to that of a nitroxide monoradical and contains three hyperfine lines with isotropic hyperfine splitting A_iso_ = 1.49(2) mT, centered at isotropic g-value g_iso_ = 2.0063(2), with a hyperfine line width (Δ*H_PP_*) of ~0.12 mT (Figure 5). The RT X-band EPR spectra prove that in biradical **2**, exchange coupling *J* is negligible, despite the relatively short distance between the two radical centers (1.48 nm). The obtained values are in good agreement with the geometry that follows from the X-ray structure, where directions of the two N–O bonds are almost parallel, and two out-of-plane vectors **z** of the nitroxide moieties are perpendicular to each other and to the spin–spin connecting vector. Therefore, biradical **2** possesses optimized geometry for DNP [67].

**Scheme 3:**
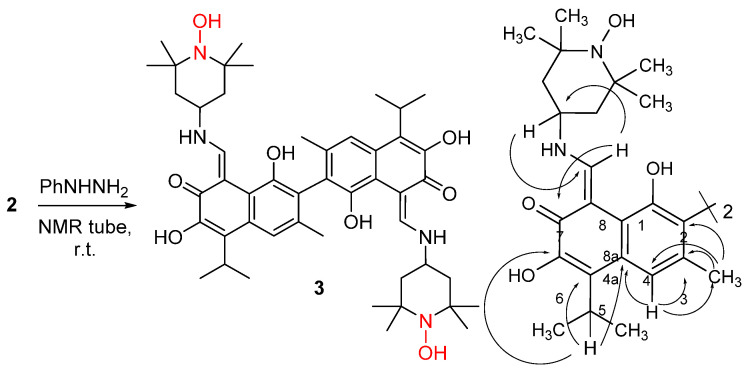
The preparation of diamagnetic derivative **3** and observed interactions in its HMBC spectrum.

For further analysis of gossypol nitroxide **2** in solution, it was reduced by treatment with phenylhydrazine into corresponding *bis*-hydroxylamine **3**. In ^1^H NMR spectra of derivative **3** in various solvents, signals of the protons of hydroxyl groups and the signals of NH groups are well separated, and their chemical shifts are independent of the concentration used. This finding means that all protons of these groups participate in the formation of intramolecular hydrogen bonds, and Schiff base **3** exists as monomers in solutions. Furthermore, NMR spectra suggest that in solution, **3** exists in the enamine–enamine form, as evidenced by the presence (in the ^1^H NMR spectrum) of a signal of a CH group at 8.8 ppm and (in the ^13^C NMR spectrum) of signals of two carbon atoms at 149 and 170 ppm. Note that after these experiments, complete assignment of the signals present in the ^1^H and ^13^C NMR spectra was carried out using two-dimensional C-H correlation spectra HMQC and HMBC (Scheme 3).

An assessment of the antiproliferative properties of compound **2** in comparison with starting gossypol (**1**) was performed on K-562 (human chronic myeloid leukemia), A-549 (human lung adenocarcinoma), HCT-116 (human colon adenocarcinoma), HEK293 (human embryonic kidney cells), and hFB-hTERT6 (non-malignant human skin fibroblasts) cell lines by the MTT assay [68]. Selected cancer cell lines are examples of adherent or suspension tumor cell lines that can show dramatic differences in sensitivity to potential chemotherapeutic compounds, while non-malignant lines are widely used to screen for compounds that selectively act on the growth of malignant cells [69]. Doxorubicin was used as a reference drug to confirm the reliability of the results of the MTT test (positive control). This antitumor antibiotic is widely used as the “gold standard” in anticancer screening both in vitro and in vivo [70,71].

Our screening results revealed that the obtained gossypol nitroxide **2** suppresses tumor cell growth at micromolar concentrations (Table 3). Nonetheless, in comparison with gossypol **1**, antiproliferative potency of conjugate **2** was considerably lower. It should also be noted that solid-tumor cell lines (A-549 and HCT-116) were noticeably less sensitive to gossypol **1** and its nitroxide **2** than leukemia cells (K-562) were. At the same time, the cytotoxicity of gossypol nitroxide **2** and paternal gossypol **1** for normal fibroblasts and for non-malignant HEK293 cells was comparable to the potency to malignant cells, which indicates that the cytotoxic effect of gossypol and its derivative **2** is not selective. The IC_50_ values for doxorubicin for all cell lines were in the submicromolar range, which corresponds to the literature data [70,71] and confirmed the reliability of the screening results.

Thus, conjugation of gossypol with the nitroxide radical significantly reduces its cytotoxic properties, thereby possibly leading to a decrease in the side effects characteristic of this natural polyphenol. This finding indicates good prospects for further in-depth evaluation of biological properties of the obtained derivative **2**, for example, for the development of new contrast reagents (for magnetic resonance imaging methods), antioxidant agents, or antiviral drugs.

## 3. Materials and Methods

### 3.1. General

Racemic gossypol was extracted from seeds of cotton (*Gossypium herbaceum*) according to the procedure given in Ref. [72]. The purity of this compound was verified by means of ^1^H NMR spectra. 4-Amino-TEMPO was synthesized from commercially available 4-amino-2,2,6,6-tetramethylpiperidine according to known procedures [73]. Methanol and acetone were distilled under atmospheric pressure prior to use without additional dehydration. Methylene chloride was washed with a 20% aqueous solution of NaOH, dried with CaCl_2_ overnight, and distilled over phosphorus pentoxide.

NMR spectra were recorded on a Bruker AC-200 spectrometer (Bruker Corporation, Billerica, MA, USA). Two-dimensional HMQC and HMBC spectra were recorded with the help of a Bruker Avance III 500 instrument (Bruker Corporation, Billerica, MA, USA). Mass spectra (electrospray ionization; ESI) were acquired on a Bruker maXis Impact spectrometer (Bruker Corporation, Billerica, MA, USA) at a capillary potential of 4500 V, with direct (syringe) injection of each sample as a solution in methylene chloride (3 μL/min). An IR spectrum was determined on a Bruker Alfa III instrument (Bruker Corporation, Billerica, MA, USA) in a KBr pellet (1.5 mg/200 mg). EPR spectra were acquired on a Jeol JES-FA200 instrument (Akishima, Tokyo, Japan) at RT in a dilute (10^−4^ M) toluene solution degassed via bubbling of argon (spectrometer settings: frequency, 9.87 GHz; microwave power, 0.5 mW; modulation amplitude, 0.05–0.15 mT; time constant, 20.5 ms; and conversion time, 20 ms). The isotropic g-value was measured experimentally using MgO doped with Mn(II) ions as a standard, placed in the resonator simultaneously with the solution under study. Melting points were measured on a Boetius apparatus and are uncorrected. Reactions were monitored by thin-layer chromatography (TLC) on Merck 60 F254 UV-254 plates (Merck KGaA, Darmstadt, Germany).

### 3.2. Synthesis of (8Z,8′Z)-1,1′,6,6′-Tetrahydroxy-8,8′-bis{[(1-oxyl-2,2,6,6-tetramethylpiperidin-4-yl)amino]methylene}-5,5′-diisopropyl-3,3′-dimethyl-2,2′-binaphthalene-7,7′(8H,8′H)-dione (***2***)

To a solution of gossypol **1** (100 mg, 0.19 mmol) in methanol (10 mL), 4-amino-TEMPO (66 mg, 0.38 mmol) was added, and the mixture was stirred until dissolution and kept at RT for 48 h. The resultant precipitate was filtered off and washed with methanol (the solubility of the product was approximately 3 mg/mL). After air drying, 95 mg (59%) of the substance was obtained as a brown powder. The substance is a solvate with methanol in a molar ratio of 1:1 (judging by NMR and elemental analyses). Additional purification was carried out by flash chromatography on SiO_2_, with elution by a gradient of n-hexane–ethyl acetate (100:0 to 60:40) and subsequent crystallization from a mixture of anhydrous acetone with dichloromethane to obtain solvent-free **2**:

**2** (red-brown), 80 mg (51%); decomposition temperature = 255 °C; *R*_f_ 0.65 (hexane/ethyl acetate at 1:1); ^1^H NMR (200 MHz, CDCl_3_) δ = 1.18 (s, 12H, NC(CH_3_)_2_), 1.23 (s, 12H, NC(CH_3_)_2_), 1.56 (d, *J* = 6.7 Hz, 6H, HC(C*H*_3_)_2_), 1.58 (d, *J* = 6.8 Hz, 6H, HC(C*H*_3_)_2_), 1.86 (d, *J* = 12.0 Hz, 4H, CH_2_), 2.00 (dd, *J* = 3.0, 12.0 Hz, 4H, CH_2_), 2.16 (s, 6H, H_3_C-C(3,3′)), 3.72 (hept, *J* = 6.8 Hz, 2H, *H*C(CH_3_)_2_), 3.7–4.0 (m, 2H, NCH), 7.64 (s, 2H, HC(4, 4′)), 9.76 (d, *J* = 11.7 Hz, 2H, HC=), 13.46 (s, 1H, NH)). IR (KBr, cm^−1^): 1178, 1241, 1308, 1362, 1431, 1495, 1537, 1618, 2873, 2934, 2974, 3292, 3486. UV/vis (MeCN, λ_max_/nm (lg ε)): 247 (4.6), 402 (4). HRMS (ESI): found m/z 825.4780 [M + H]^+^; calculated for C_48_H_65_N_4_O_8_^+^ 825.4797. Found (%): C, 68.60; H, 7.77; N, 6.64. C_48_H_64_N_4_O_8_·CH_3_OH. Calculated (%): C, 68.67; H, 7.82; N, 6.54. Found (%): C, 69.60; H, 7.77; N, 6.68. C_48_H_64_N_4_O_8_. Calculated (%): C, 69.88; H, 7.82; N, 6.79.

NMR spectra from the product of reduction of **2** by PhNHNH_2_. ^1^H NMR (500 MHz, CDCl_3_) δ = 13.53–13.33 (m, 2H, NH), 9.73 (d, *J* = 12.3 Hz, 2H, =CHN), 8.06 (s, 2H, HO-C(6, 6′)), 7.64 (s, 2H, HC(4, 4′)), 5.72 (brs, 2H, HO-C(1, 1′)), 3.73–3.78 (m, 2H, *H*C(CH_3_)_2_), 3.69–3.73 (m, 2H, HCN), 2.14 (s, 6H, H_3_C-C(3, 3′)), 1.95 (ddd, *J* = 12.5, 10.2, 3.7 Hz, 4H, HC-e(3, 3′, 5, 5′)), 1.75 (t, *J* = 12.5 Hz, 4H, HC-a(3, 3′, 5, 5′)), 1.55 (d, *J* = 7.3 Hz, 6H, (*H*_3_C)_2_CH), 1.53 (d, *J* = 7.3 Hz, 6H, (*H*_3_C)_2_CH), 1.22 (s, 12H, H_3_C-C(2, 2′, 6, 6′))), 1.18 (s, 6H, H_3_C-C(2, 2′, 6, 6′))), 1.17 (s, 6H, H_3_C-C(2, 2′, 6, 6′))). ^13^C NMR (50 MHz, CDCl_3_) δ = 172.48 (C(7, 7′)), 160.74 (=CN), 148.90 (C(1, 1′)), 147.19 (C(6, 6′)), 131.82 (C(3, 3′)), 128.33 (C(5, 5′)), 127.42 (C(4a, 4a′)), 118.24 (C(4, 4′)), 115.75 (C(2, 2′)), 114.48 (C(8a, 8a′)), 103.20 (C(8, 8′)), 58.84 (C(2″,6″)), 51.86 (C(4″)), 46.02 (C(3″, 5″)), 32.21 (*C*H_3_C(2″, 6″)), 27.45 ((CH_3_)_2_*C*), 20.38((*C*H_3_)_2_C), 20.33((*C*H_3_)_2_C), 20.08 (*C*H_3_-C(2″, 6″)), 19.82 (*C*H_3_C(2″, 6″)).

### 3.3. Crystal Structure Determination and Refinement

Crystals of derivative **2** were obtained by evaporation of a solution in a mixture of anhydrous acetone and dichloromethane. X-ray diffraction data were collected at 100 K on a four-circle Rigaku Synergy S diffractometer equipped with a HyPix6000HE (Rigaku Holdings Corporation, Japan) area detector (kappa geometry and shutterless ω-scan technique), using monochromatized Cu K_α_ radiation. The intensity data were integrated and corrected for absorption and decay by means of the CrysAlisPro software (Version 1.171.41) [74]. The structure was solved by direct methods using SHELXT [75] and refined on *F*^2^ using SHELXL-2018 [76] in the OLEX2 program [77]. All nonhydrogen atoms were refined with individual anisotropic displacement parameters. Locations of amino and hydroxyl hydrogen atoms were determined in the electron density difference map; these hydrogen atoms were refined with individual isotropic displacement parameters. All other hydrogen atoms were placed at ideal calculated positions and refined as riding atoms with relative isotropic displacement parameters. A rotating group model was applied to methyl groups. Crystal data and structure refinement for **2** are given in Table 4.

### 3.4. Antiproliferative Activity

The K-562 (human chronic myeloid leukemia cells), A-549 (human lung adenocarcinoma), HCT-116 (human colon adenocarcinoma), and HEK293 (human embryonic kidney cells) cell lines used in the experiment were purchased from the American Type Culture Collection (ATCC, Manassas, VA, USA). Cell line hFB-hTERT6 (non-malignant human skin fibroblasts) was obtained via the lentiviral transduction of full-length TERT genes under a cytomegalovirus promoter (generated at Engelhardt Institute of Molecular Biology, Moscow, by Dr. E. Dashinimaev; gift of Prof. A. Shtil). Adherent cells (HCT-116 and A-549) were cultured in Dulbecco’s modified Eagle’s medium (DMEM, PanEco, Russia) supplemented with 10% of fetal calf serum (HyClone-Cytiva, Logan, UT, USA), 2 mM L-glutamine, 100 U/mL penicillin, and 100 μg/mL streptomycin at 37 °C and 5% CO_2_ in a humidified atmosphere. Suspension cells (K-562) were cultured in RPMI-1640 (PanEco, Russia) with the same supplements. Chemotherapeutic drug doxorubicin (Sigma-Aldrich, St. Louis, MO, USA) was used as a positive control. The cytotoxicity was determined in a formazan assay (MTT assay). The tested compounds were dissolved in DMSO to a concentration of 10 mM. The cells (5 × 10^3^ in 190 μL of the culture medium) were seeded in a 96-well plate (Nalge NUNC International, Rochester, NY, USA) and treated with the test compounds (0.10–50.00 μM; each concentration was tested as three biological replicates), doxorubicin (positive control), or 0.1% DMSO (vehicle control). The treated cells were incubated for 72 h at 37 °C and 5% CO_2_ in a humidified atmosphere. The cells in the logarithmic growth phase were subjected to the experiment. The cells without the drugs served as a control.

After incubation, 20 μL of an aqueous MTT solution (3-(4,5-dimethylthiazol-2-yl)-2,5-diphenyltetrazolium bromide, Sigma-Aldrich, St. Louis, MO, USA; 5 mg/mL) was added into each well of the plate and incubated for another 2–3 h. Media were removed from the cells. Suspension cells (K-562) were precentrifuged in a plate centrifuge (Thermo Fisher Scientific, Waltham, MA, USA) for 10 min at 2000 rpm. The resulting formazan precipitate was dissolved in DMSO, and absorption was measured at 570 nm. Cytotoxic efficiency was calculated as the percentage of absorption in the wells containing cells treated with a drug relative to the control cells (100%). Based on the results of the calculations, survival curves were constructed for all the compounds evaluated in the MTT assay, and IC_50_ was determined: the concentration of the compound that inhibited cell growth by 50%.

## 4. Conclusions

We succeeded in the synthesis of the first derivative of gossypol with stable paramagnetic functions: a Schiff base of gossypol with two molecules of amino-TEMPO. The molecular and crystal structure of the conjugate was determined by X-ray diffraction. It was revealed that in crystals, gossypol nitroxide **2** exists in an enamine–enamine tautomeric form. The tautomer is strongly stabilized by intra- and intermolecular hydrogen bonds promoted by the resonance of π-electrons in the aromatic system. NMR analyses of gossypol derivative **2** proved that in solutions, the enamine–enamine tautomeric form prevails. RT X-band EPR spectra indicate that the *J* exchange coupling in the rigid biradical with the gossypol framework is negligible, and the two nitroxide moieties are almost orthogonal, which is optimal for efficient solid-state DNP enhancements. Additionally, gossypol nitroxide hybrid compound **2** was tested for its antiproliferative properties. It was noted that at micromolar concentrations, gossypol nitroxide hybrid **2** suppresses the growth of tumor cell lines of different origins. Nevertheless, compared to parental gossypol **1**, the antiproliferative activity of gossypol nitroxide **2** is noticeably lower, and therefore, this type of modification of this natural compound could be used for decreasing the strong cytotoxicity of such compounds.

We believe that this new avenue of the synthesis and investigation of open-shell molecules derived from gossypol is promising for the search for new pharmaceutics. Within this field, compounds with a new set of pharmacological characteristics can be obtained, as can new contrast reagents for magnetic resonance imaging methods, antioxidants with combined actions, and rigid two-spin systems for DNP. It should also be mentioned that either identical or different radical groups can be introduced into such a polyfunctional molecule, and the arsenal of these groups is currently diverse. Furthermore, we find it interesting to synthesize gossypol derivatives that together with paramagnetic functions will also contain pharmacophores.

## Figures and Tables

**Table 1:** Selected bond lengths (Å) and angles (°) in the X-ray structures of **2**.

Bond Lengths			
O(1)–C(6)	1.3549(15)	C(8)–C(25)	1.4135(18)
O(2)–C(7)	1.2765(15)	C(9)–C(10)	1.4142(17)
O(3)–C(10)	1.3684(14)	C(13)–O(7B)	1.346(3)
O(4)–N(2)	1.2903(17)	C(14)–C(15)	1.4554(16)
O(5)–C(16)	1.3656(14)	C(14)–C(19)	1.4256(17)
O(6)–C(17)	1.2884(15)	C(15)–C(16)	1.3641(18)
O(8)–N(4)	1.2933(16)	C(16)–C(17)	1.4440(18)
N(1)–C(25)	1.3164(16)	C(17)–C(18)	1.4230(17)
N(1)–C(26)	1.4575(17)	C(18)–C(19)	1.4573(17)
N(3)–C(39)	1.3124(17)	C(18)–C(39)	1.4205(18)
N(3)–C(40)	1.4621(17)	C(20)–O(7A)	1.3577(15)
C(1)–C(10)	1.3912(17)	C(4)–C(9)	1.4274(17)
C(1)–C(11)	1.4955(16)	C(5)–C(6)	1.3619(17)
C(2)–C(3)	1.3812(17)	C(6)–C(7)	1.4475(18)
C(3)–C(4)	1.4124(17)	C(7)–C(8)	1.4223(17)
C(4)–C(5)	1.4555(17)	C(8)–C(9)	1.4609(16)
Bond angles			
C(6)–O(1)–H(1)	108.1(14)	O(5)–C(16)–C(17)	115.24(11)
C(10)–O(3)–H(3)	112.1(13)	C(15)–C(16)–O(5)	121.05(11)
C(16)–O(5)–H(5)	105.9(14)	O(6)–C(17)–C(16)	116.94(11)
C(25)–N(1)–H(1A)	117.6(12)	O(6)–C(17)–C(18)	123.82(12)
C(25)–N(1)–C(26)	124.77(12)	C(39)–C(18)–C(19)	123.99(11)
C(26)–N(1)–H(1A)	116.8(12)	C(20)–C(19)–C(18)	123.44(11)
C(39)–N(3)–H(3A)	111.5(13)	O(7A)–C(20)–C(19)	117.71(11)
C(39)–N(3)–C(40)	125.36(11)	C(5)–C(22)–C(23)	111.54(11)
C(40)–N(3)–H(3A)	123.2(13)	N(1)–C(25)–C(8)	123.45(12)
O(1)–C(6)–C(5)	121.76(11)	N(1)–C(26)–C(27)	109.69(12)
O(1)–C(6)–C(7)	115.61(11)	N(1)–C(26)–C(30)	109.36(12)
O(2)–C(7)–C(6)	117.03(11)	N(3)–C(39)–C(18)	123.15(12)
O(2)–C(7)–C(8)	123.52(12)	N(3)–C(40)–C(41)	110.85(11)
O(3)–C(10)–C(1)	119.65(11)	N(3)–C(40)–C(44)	107.55(11)
O(3)–C(10)–C(9)	117.21(10)	C(20)–O(7A)–H(7A)	109.5

**Table 2:** Dimensions of the hydrogen bonds (Å and °) within the crystal structure of gossypol imine **2**.

D–H⋯A	*d*(D–H)	*d*(H⋯A)	*d*(D⋯A)	∠(DHA)
O(1)–H(1)⋯O(2)	0.84(2)	2.12(2)	2.6146(14)	117.7(17)
O(1)–H(1)⋯O(2) ^i^	0.84(2)	1.93(2)	2.6609(13)	145.7(19)
O(3)–H(3)⋯O(6) ^ii^	0.88(2)	1.92(2)	2.6367(12)	137.7(19)
O(5)–H(5)⋯O(3) ^ii^	0.95(2)	2.11(3)	3.0209(14)	160(2)
O(5)–H(5)⋯O(6)	0.95(2)	2.03(2)	2.6115(14)	117.8(18)
N(1)–H(1A)⋯O(2)	0.95(2)	1.78(2)	2.5314(15)	133.6(17)
N(3)–H(3A)⋯O(6)	0.90(2)	1.76(2)	2.5400(15)	143.8(19)

Symmetry transformations that were utilized to generate equivalent atoms: (i) −x + 1/3, −y + 2/3, and −z + 2/3; (ii) −x + 1, −y + 1, and −z + 1.

**Table 3:** Antiproliferative activity (half-maximal inhibitory concentration: IC_50_, μM) of gossypol derivative **2** in comparison with original gossypol **1** and doxorubicin.

Compound	IC_50_ *, μM				
	K-562	A-549	HCT-116	HEK293	hFB-hTERT6
**2**	15.5 ± 2.2	31.2 ± 3.2	28.2 ± 3.9	30.2 ± 3.3	16.3 ± 2.1
Gossypol **1**	0.80 ± 0.11	4.8 ± 0.4	5.3 ± 0.6	2.8 ±0.3	3.0 ± 0.4
Doxorubicin	0.10 ± 0.01	0.35 ± 0.04	0.42 ± 0.02	0.15 ± 0.02	0.12 ± 0.01

* The concentration of a compound that inhibits cell growth by 50%; results are presented as the mean of three independent experiments ± standard deviation.

**Table 4:** Crystal data and structure refinement for **2**.

Empirical formula	C_48_H_64_N_4_O_8_
Formula weight	825.03
Temperature	99.98(11) K
Wavelength	1.54184 Å
Crystal system, space group	Trigonal, *R*-3
Unit cell dimensions	*a* = 37.2827(3) Å
	*b* = 37.2827(3) Å
	*c* = 20.4854(2) Å
Angles	α = 90°
	β = 90°
	γ = 120°
Volume	24,659.8(5) Å^3^
Z	18
Density (calculated)	1.000 g/cm^3^
Absorption coefficient	0.546 mm^−1^
F(000)	7992
Crystal size	0.15 × 0.13 × 0.09 mm^3^
Theta range for data collection	2.370° to 79.875°
Index ranges	−43 ≤ h ≤ 47, −43 ≤ k ≤ 46, −25 ≤ l ≤ 26
Reflections collected	55,651
Independent reflections	11,783 [R(int) = 0.0270]
Completeness to theta = 67.684°	99.9%
Absorption correction	Analytical
Max. and min. transmission	0.998 and 0.997
Refinement method	Full-matrix least-squares on F^2^
Data/restraints/parameters	11,783/9/587
Goodness of fit on *F*^2^	1.044
Final R indices [*I* > 2sigma(*I*)]	*R*_1_ = 0.0478, w*R*_2_ = 0.1283
R indices (all data)	*R*_1_ = 0.0525, w*R*_2_ = 0.1317
Largest diff. peak and hole	0.421 and −0.285 e·Å^−3^

## Data Availability

The data and supporting information that are necessary to reproduce our results are available within the article. The raw data are available upon request.

## Supplementary Materials

The following supporting information can be downloaded at: https://www.mdpi.com/article/10.3390/molecules29204966/s1, Figure S1: ^1^H NMR (200 MHz) spectrum of radical **2** at presence of PhNHNH_2_; Figure S2: ^1^H NMR (500 MHz) spectrum of **3** at presence of PhNHNH_2_; Figure S3: ^13^C NMR (50 MHz) spectrum of **3** at presence of PhNHNH_2_ (signals at 112.16, 119.59, 129.23 and 151.19 ppm belong to PhNHNH_2_); Figure S4: HMQC NMR spectrum of **3**; Figure S5: HMBC NMR spectrum of **3**; Figure S6: IR spectrum of compound **2** in a KBr pellet; Figure S7: HRMS of biradical **2**.
