# Supplementary material for: A Spin-Labeled Derivative of Gossypol
Source: Molecules. 2024 Oct 21;29(20):4966. doi: 10.3390/molecules29204966 (PMC11510377; doi:10.3390/molecules29204966)
Supplement: Supplementary file 1 [file molecules-29-04966-s001.zip › molecules-3244604-supplementary.pdf]

Supplementary materials to the article:

A spin-labeled derivative of gossypol

Andrey V. Stepanov, Vladimir N. Yarovenko, Darina I. Nasyrova, Lyubov G. Dezhenkova, Igor O. Akchurin, Mickhail M. Krayushkin, Valentina V. Ilyushenkova, Andrey E. Shchekotikhin and Evgeny V. Tretyakov.

NMR spectra were recorded to 2 mg/ml solutions in $CDCl_3$ and should be recorded as quick as possible due to slow reaction of hydroxylamine **3** with $PhNHNH_2$.

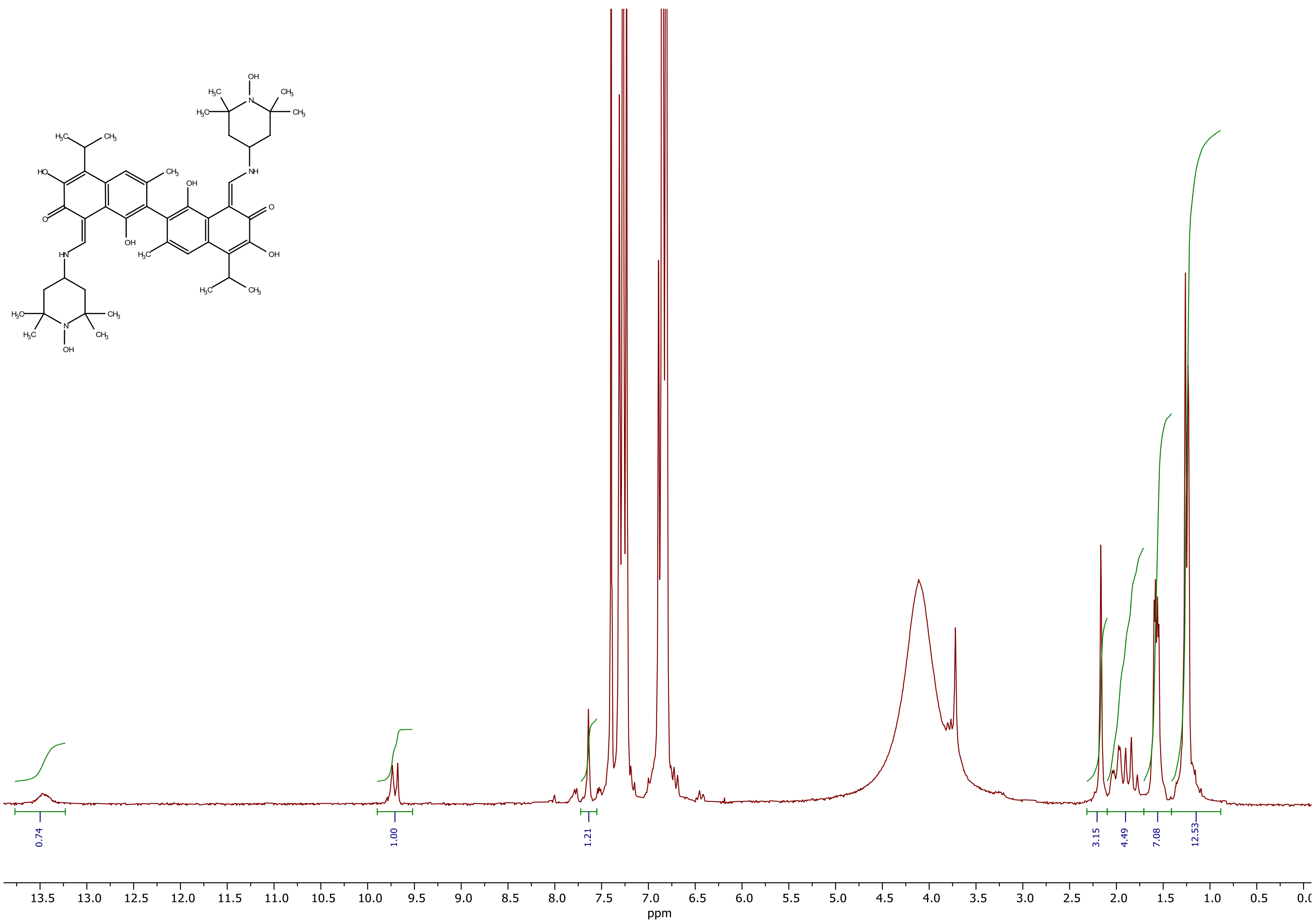

Figure S1. ^{1}H NMR (200 MHz) spectrum of radical **2** at presence of $PhNHNH_2$.

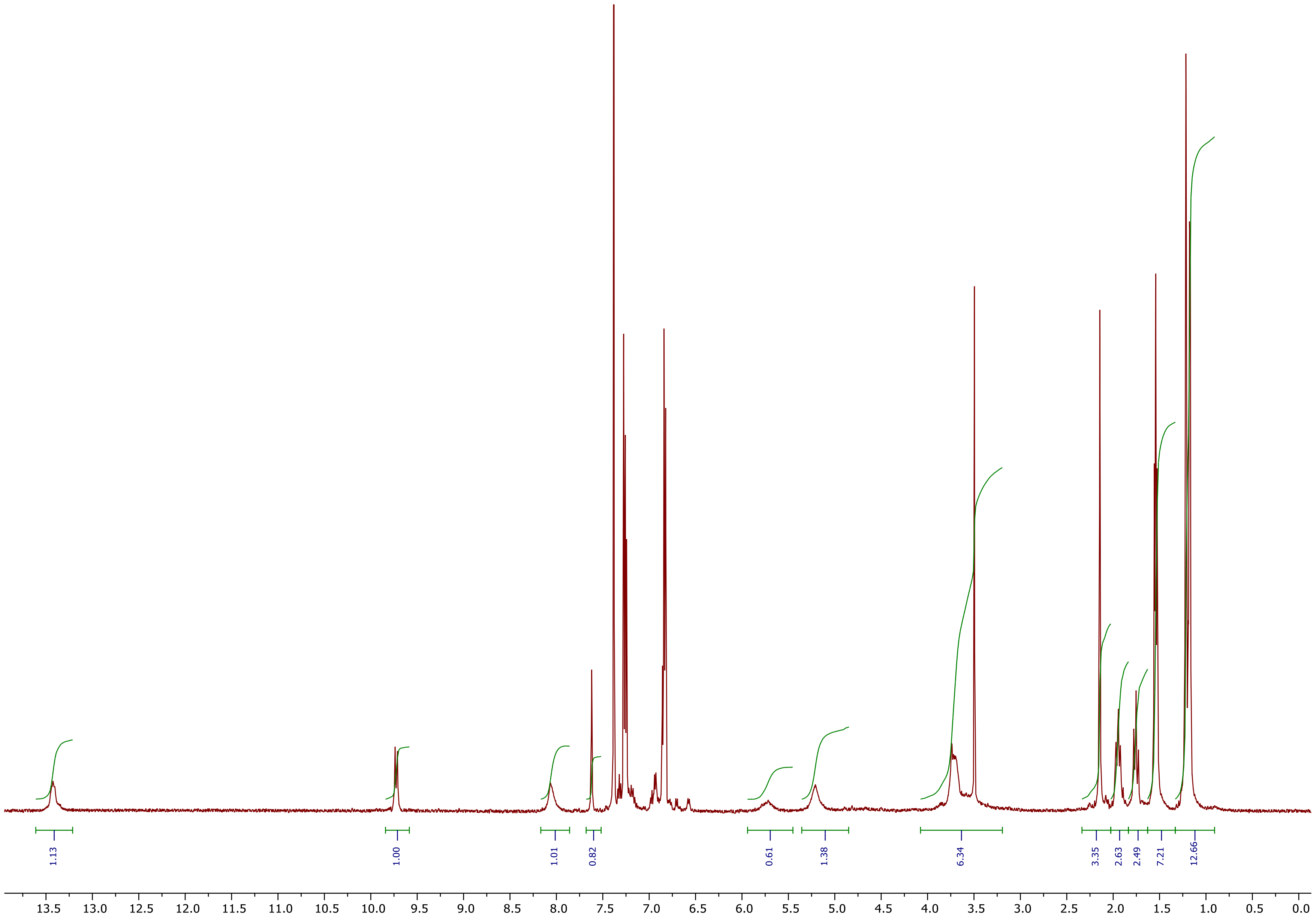

Figure S2. ^{1}H NMR (500 MHz) spectrum of **3** at presence of $PhNHNH_2$.

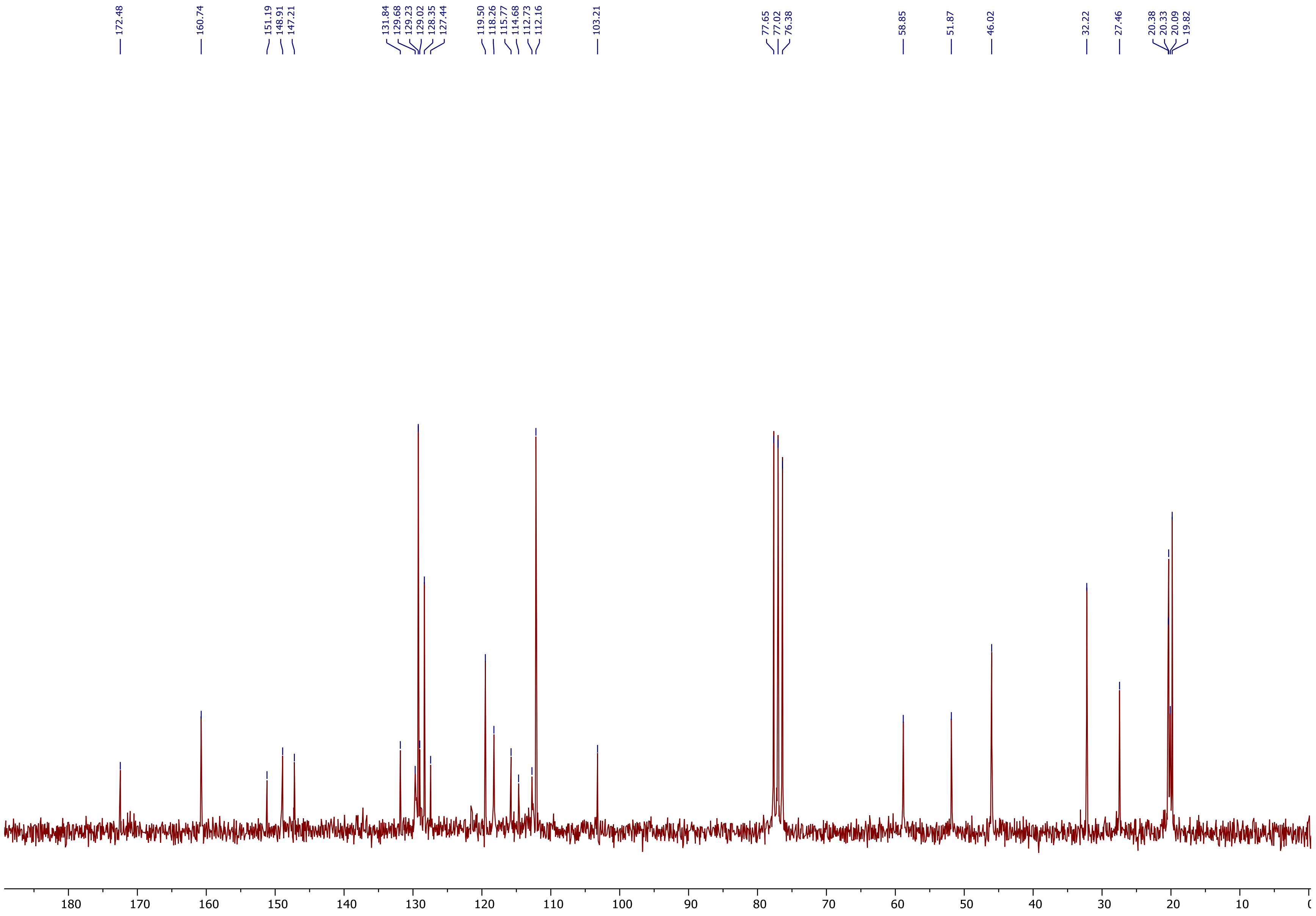

Figure S3. ^{13}C NMR (50 MHz) spectrum of **3** at presence of $PhNHNH_2$ (signals at 112.16, 119.59, 129.23 and 151.19 ppm belong to $PhNHNH_2$).

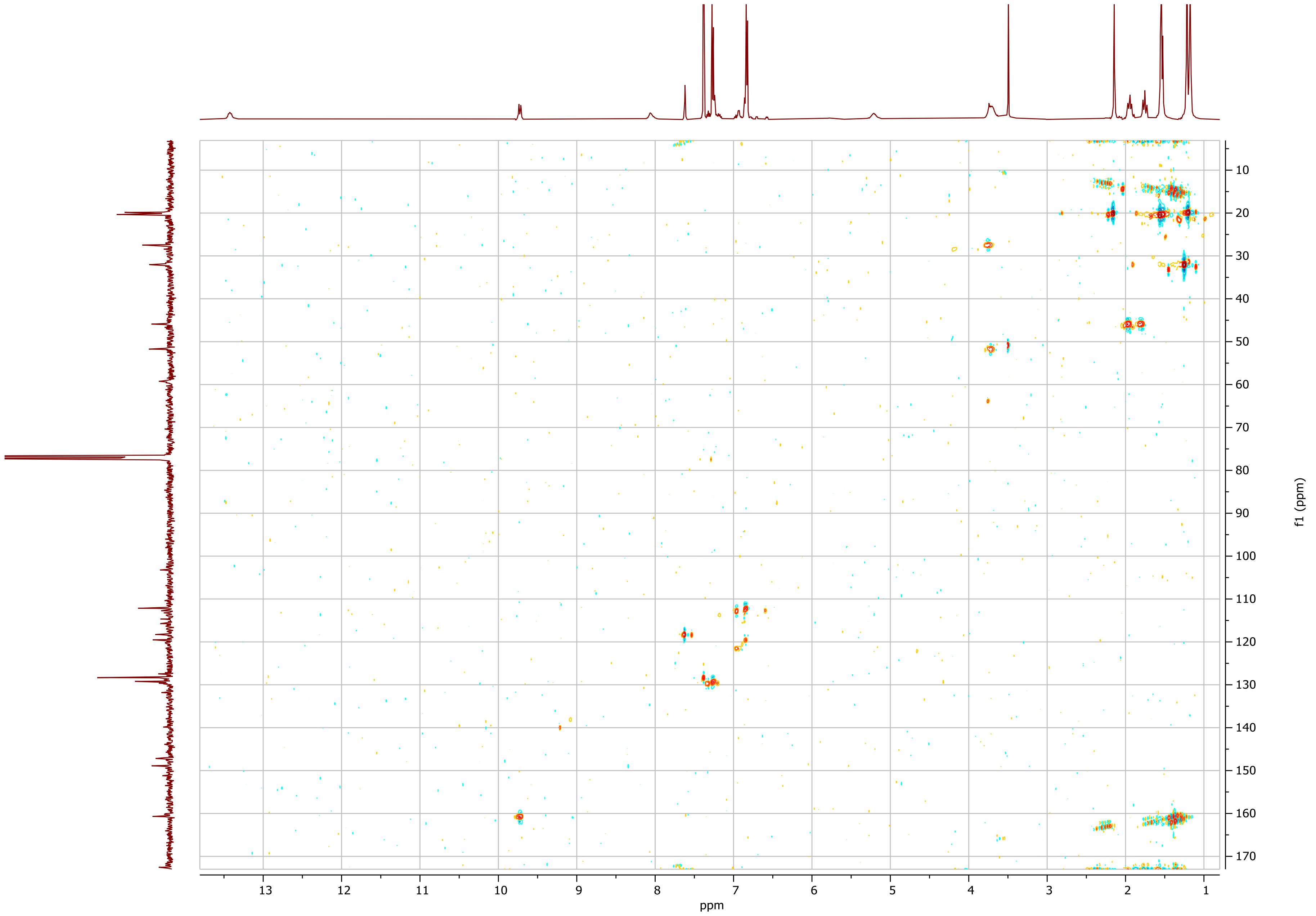

Figure S4. HMQC NMR spectrum of **3**.

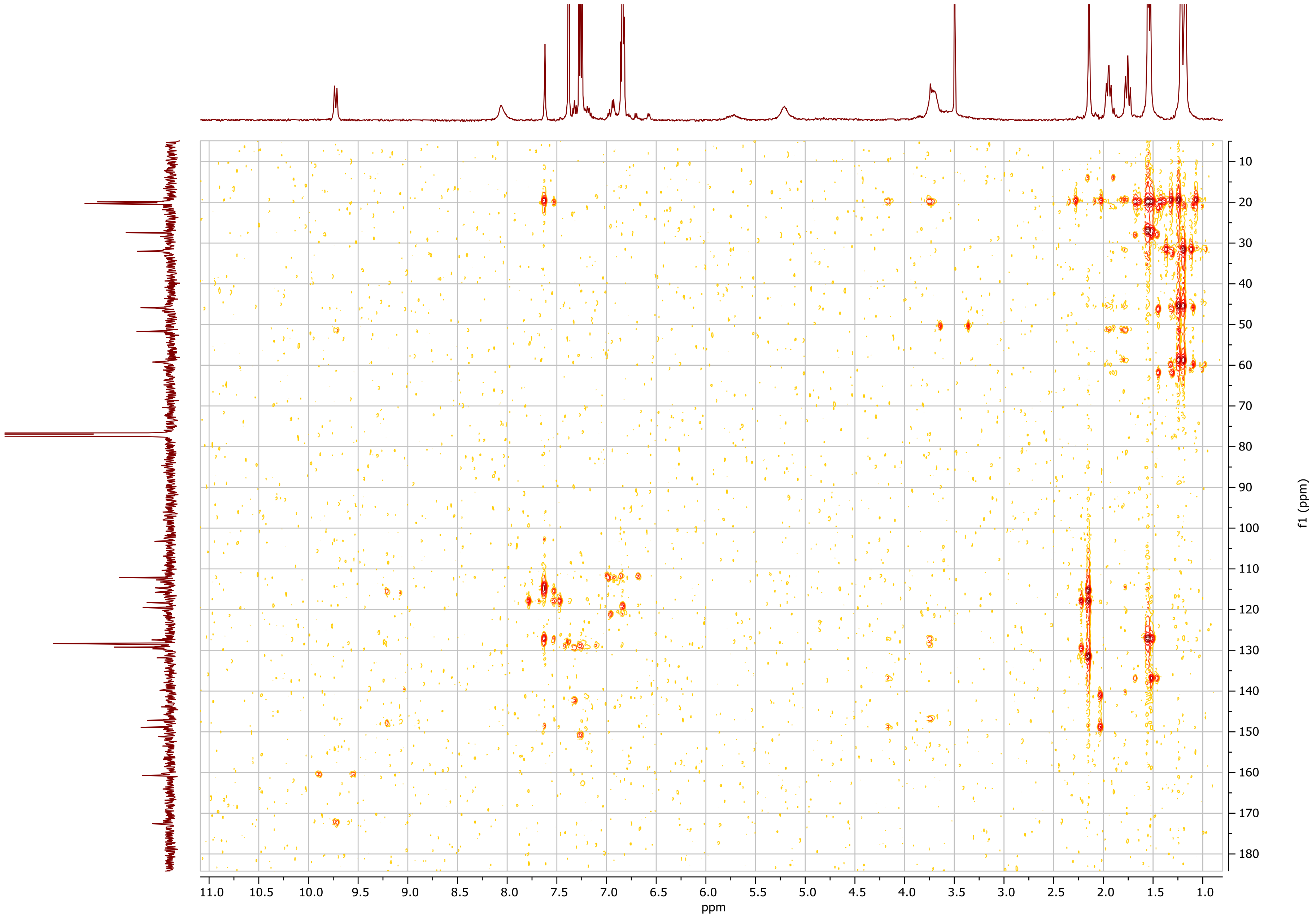

Figure S5. HMBC NMR spectrum of **3**.

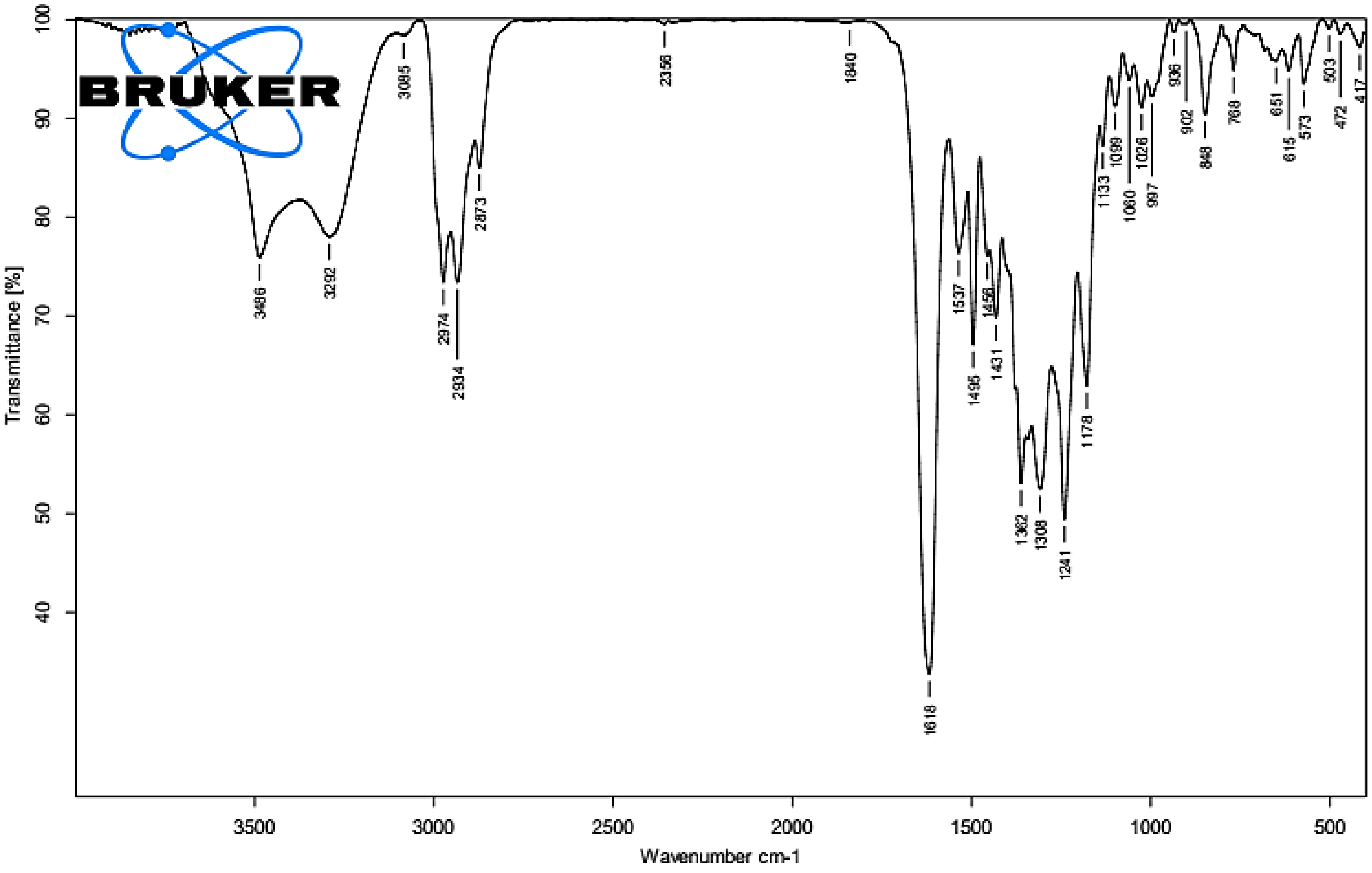

Figure S6. IR spectrum of compound **2** in a KBr pellet.

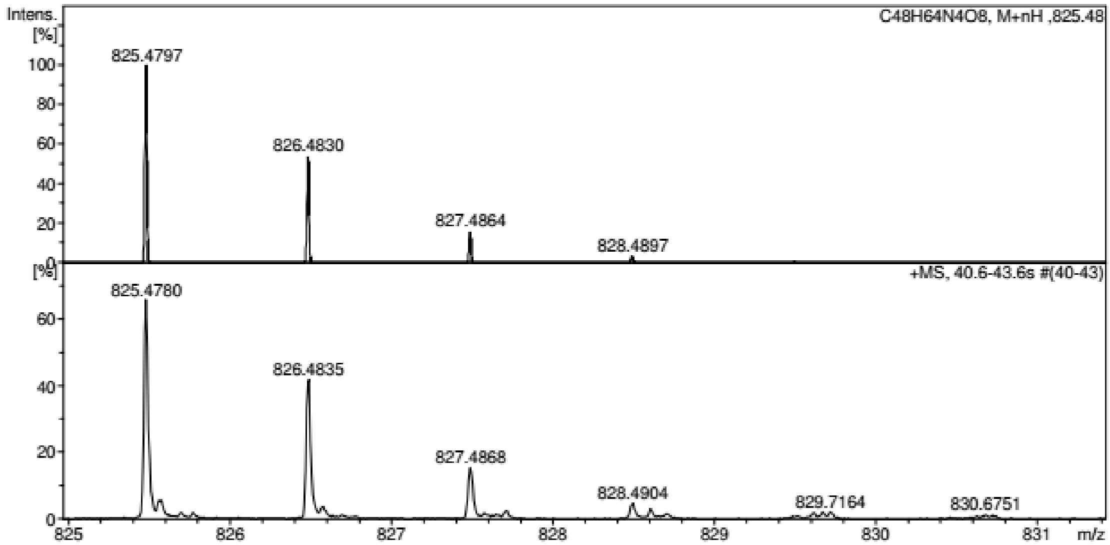

Bruker Compass DataAnalysis 4.0 printed: 26.01.2024 18:16:19 Page 1 of 1

Figure S7. HRMS of biradical **2**.
